# Rosemary Ethanolic Extract-Loaded Electrospun Nanofibers as a Bioactive Dressing for Diabetic Wound Healing

**DOI:** 10.7150/ntno.130423

**Published:** 2026-10-09

**Authors:** Rachmat Mauludin, Bintang Nurrani, Afrillia Nuryanti Garmana, Muhammad Miftahul Munir

**Affiliations:** 1 Department of Pharmaceutics, School of Pharmacy, Institut Teknologi Bandung, Bandung 40132, Indonesia.; 2 Department of Pharmacology and Clinical Pharmacy, School of Pharmacy, Institut Teknologi Bandung, Bandung 40132, Indonesia.; 3 Department of Physics, Faculty of Mathematics and Natural Sciences, Institut Teknologi Bandung, Bandung 40132, Indonesia.

**Keywords:** antibacterial, antioxidant, diabetic wound, nanofiber, rosemary extract

## Abstract

Diabetic wounds are characterized by dysregulated reactive oxygen species (ROS), bacterial colonization, and excessive wound exudate. Current chronic wound management follows the TIME framework, in which infection-inflammation control and moisture balance can be addressed through wound dressings. Electrospun nanofibers incorporating plant-derived bioactives, such as rosemary ethanolic extract, offer antioxidant, antibacterial, and pro-healing properties relevant to diabetic wound therapy. In this study, rosemary ethanolic extract-loaded nanofibers were fabricated via electrospinning at 14 kV and 75% relative humidity, with a spinneret-collector distance of 13 cm, a flow rate of 1 mL/h, and a spinneret diameter of 0.83 mm. The pre-electrospinning formulation of nanofibers was evaluated for antibacterial activity by agar diffusion, also nanofiber evaluated for antioxidant activity using the DPPH radical scavenging assay and microbial penetration using *Staphylococcus aureus* and *Staphylococcus epidermidis*, and wound closure in streptozotocin-induced mice. The resulting nanofibers exhibited smooth morphology, a green appearance, and an average diameter of 237.51 ± 39.37 nm. Although the nanofibers exhibited an initial hydrophobic surface, they exhibited a high swelling capacity (481.09 ± 4.69%) indicating that water was gradually absorbed into the porous matrix and enabling substantial exudate absorption. Encapsulation efficiency was 86.17 ± 3.02%, and release kinetics followed the Korsmeyer-Peppas model. The rosemary extract-loaded formulation contained 2.067 ± 0.004 mg ascorbic acid equivalent per gram and produced inhibition zones of 20.60 ± 1.65 mm against *S. aureus* and 19.37 ± 0.64 mm against *S. epidermidis*. The electrospun nanofibers effectively prevented microbial penetration at 32°C and 37°C. *In vivo*, the wound-closure area under the curve (AUC) was significantly greater than that of the 0.9% sodium chloride control. Both unloaded cellulose acetate nanofibers and rosemary ethanolic extract-loaded cellulose acetate nanofibers promoted diabetic wound healing compared with the sodium chloride control. Although the rosemary extract-loaded nanofiber showed favorable wound healing outcomes, the present *in vivo* study did not clearly demonstrate an additional therapeutic benefit of rosemary ethanolic extract over the unloaded nanofiber.

## Introduction

Diabetic wound, a type of chronic wound, can be treated through chronic wound therapy, which applies the TIME principle that includes tissue debridement, infection control, moisture balance control, and evaluation of wound edges. Debridement is the removal of dead cells through surgical procedures. The next two components, infection and inflammation control and moisture control, can be performed with wound dressings, which can use a variety of materials, including nanofibers 1. Impregnating wound dressings with medicines can enhance wound healing. Therefore, incorporating plant extracts to nanofibers can improve diabetic wound healing, as reflected in their good physical characteristic, excellent antibacterial activity, and promising results from *in vivo* evaluation 2.

Nanofibers are among 1D nanomaterials with many controllable characteristics, that can be used for diabetic wound healing 3. Nanofibers' viability for enhancing wound healing owe to their flexibility, good mechanical properties, and controllable contained agents. Nanofibers are able to mimic extracellular matrix and can be fabricated through electrospinning, showing several advantageous characteristics, such as high porosity, ease to fabricate, and the ability to inhibit microbial penetration 2, 3.

Electrospun nanofibers incorporating plant-derived bioactive compounds have been extensively explored for diabetic wound therapy. Rosemary (*Rosmarinus officinalis*) represents a promising candidate, available in both essential oil and extract forms; however, its extract has received comparatively limited development despite its therapeutic potential 4, 5, 6. Rosemary extract contributes to wound repair by enhancing epithelialization, accelerating wound closure, promoting neovascularization, and exerting antibacterial activity that helps restore microbial balance at the wound site 7. In diabetic wounds, an extended inflammatory phase driven by excessive neutrophil and macrophage activity impairs healing 8. The immunoregulatory properties of rosemary extract may mitigate this dysregulated inflammation 7. Furthermore, rosemary extract supports the proliferative phase by stimulating fibroblast proliferation, increasing collagen synthesis, and facilitating granulation tissue formation 7,9. These attributes highlight its potential as a natural therapeutic agent suitable for incorporation into nanofiber-based wound dressings, which provide structural protection during wound contraction 10. To date, no studies have reported the use of electrospun cellulose acetate nanofibers loaded with rosemary-leaf ethanolic extract (ER) for diabetic wound management. Therefore, this study developed and characterized ER-loaded nanofibers (NFER) and evaluated their therapeutic performance in diabetic wound healing, with the expectation that such an approach would enhance healing outcomes and offer a promising alternative treatment strategy.

## Materials and Methods

### Materials

The materials used in this research included rosemary crude plants (from Bumi Herbal Dago, Bandung), cellulose acetate (Mn~30.000 from Sigma-Aldrich), acetone (from EMSURE®), N-N-dimethylacetamide (DMAc, from Sigma-Aldrich), 2-2-diphenyl-1-picylhydrazyl (from Supelco), and Mueller-Hinton agar (from Millipore®).

### Methods

#### Rosemary Extraction

Rosemary crude plant extraction was carried out by the maceration method with 96% ethanol for three days. In the first six hours, the maceration flask was stirred occasionally. After 24 hours, the macerate was isolated up to three times. The liquid extract obtained was then dried with a rotary evaporator.

#### Phytochemical Screening

Flavonoid, saponin, tannin, steroid, terpenoid, and alkaloid screenings followed the method by Dubale *et al*. (2023) 11, while quinon and coumarine screenings followed the method by Shaikh and Patil (2020) 12.

#### Nanofiber Fabrication

Nanofiber fabrication was carried out by solving 10% w/w ER (toward cellulose acetate) and 12% w/v cellulose acetate (toward solvent) in 12 mL of acetone-DMAc mixture (v/v). The electrospinning was set with a voltage of 14 kV, a spinneret-to-collector distance of 13 cm, a flow rate of 1 mL/hour, a relative humidity of 75%, and a collection time of three hours for a thickness of ± 100 µm.

#### Nanofiber Characterization

##### Organoleptic Properties and Thickness

The organoleptic properties of the nanofibers were evaluated based on the physical appearance (i.e. the color and texture). While the thickness of the nanofibers was measured **three times** using a micrometer.

##### Morphology and Particle Diameter

The morphology of the nanofibers was evaluated using a Scanning Electron Microscope (SEM) with an accelerated voltage of 15 kV, while the particle diameter of the nanofibers was measured using ImageJ at 50 points.

##### Wettability

The wettability of nanofibers is correlated with the hydrophobicity of the nanofiber surface. Identification of the surface characteristics of nanofibers is important for maintaining bacterial growth, absorbing wound exudates, and maintaining the moisture of the skin 13, 14. The wettability of nanofibers is influenced by contact angle. In this study, testing of wettability was conducted with CAAI 2320 and myDropmeter® software. The assay began with preparing nanofibers with certain sizes, then dropping them with ~5 µL water and determining the angle between water and the nanofibers with ImageJ®.

##### Swellability

The swellability of nanofibers is correlated with the hydrophilicity of the nanofibers. This measure is essential for wound exudates absorption 14. In this study, it was evaluated using the gravimetric method with a phosphate buffer saline (PBS) solution containing 0.5% Tween 20. The quantification of swellability was carried out according to Salami *et al*. (2021) with some modifications; the procedure included weighing the nanofibers (W0), incubating them with 10 mL of PBS solution containing 0.5% Tween 20 for 24 hours, and reweighing the nanofibers (W1). The swollen nanofibers were placed on filter paper for 10 seconds to remove the adsorbed water 14.



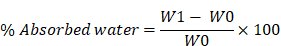



##### Encapsulation Efficiency

Encapsulation efficiency was measured with a dialysis bag according to the method by Suwantong *et al*. (2008) with some modifications. Nanofibers were weighed at 20 mg and then dissolved in 4 mL of acetone-DMAc mixture (2:1, v/v). Then, 0.5 mL of the solution was taken and added with 8 mL of PBS solution containing 0.5% Tween 20. The final solution was filtered with a nylon membrane 0.22 µm thick 15.

The quantification of encapsulation efficiency used rosmarinic acid as a marker substance. It was conducted with high-performance liquid chromatography (HPLC) according to Syarifah *et al*. (2022) with some modifications. HPLC was conducted with a Silversil® 5 µm C18 250 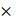
4.6 mm column and a Waters® 2487 dual 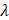
absorbance detector. The flow rate was maintained at 1 mL/min, with 30% acetonitrile and 0.1% formic acid (v/v) used as mobile phases. The UV detector was set at 330 nm 16.

##### *In Vitro* Release

The *in vitro* release of rosemary ethanolic extract from nanofibers was conducted using the method by Chuysinuan *et al*. (2017) with some modifications. This was done by placing 20 mg of nanofibers in a cellulose dialysis bag (14 kDA MWCO, Wards Science, Rochester, NY, USA) with 2 mL of PBS solution containing 0.5% Tween 20. The dialysis membrane was immersed in a chamber containing 30 mL of PBS solution containing 0.5% Tween 20. The glass vessel was placed on a magnetic stirrer, and the temperature of the solution was maintained at 37
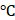
with a stirring speed of 120 rpm to maintain the sink condition. Sampling was conducted for 10, 20, 30, 40, 50, 60, 120, 240, and 360 mins, with a sampling volume of 1 mL for each sampling time. The quantification of the released rosemary ethanolic extract used rosmarinic acid as a marker substance with the same method used for encapsulation efficiency 17. Additionally, the mathematic model for the released extract from the nanofibers was determined.

#### *In Vitro* Evaluation

##### Antioxidant Activity

Antioxidant activity was measured using the method by Spasova *et al*. (2024) with some modifications. The procedure was carried out by immersing 2.5 mg of nanofibers in 3 mL of ethanolic DPPH (80 µg/mL). Evaluation was conducted on blank nanofibers (NF) and nanofibers 18.

Absorbance was determined by UV-Vis spectrophotometry at 517 nm. The absorbance of the sample with DPPH is denoted as A1, and the absorbance of DPPH alone is denoted as A0 19. Then, the antioxidant activity converted to mg ascorbic acid equivalents (AAE) and mg extract per gram of NFER or NF sample.



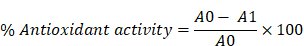



##### Antibacterial Activity

Antibacterial activity measurement was carried out on the pre-electrospinning nanofiber solution with the agar diffusion method owing to the high rigidity of the nanofibers and because low moisture hindered the penetration of substances from the nanofibers. The measurement involved *Staphylococcus aureus* and *Staphylococcus epidermidis*, whose levels are elevated in diabetes mellitus sufferers 20. The procedure for the antibacterial activity measurement followed the method by Ganesh *et al*. (2016) with some modifications. A prepared sterile MHA medium in a Petri dish was swabbed with a bacterial culture adjusted to a 0.5 McFarland standard. Then, two wells for each 10 µL of pre-electrospinning nanofiber solution, with and without rosemary extract, as well as three disc papers for each of 10 µL of 10% rosemary extract, 10 µL of acetone-DMAc mixture (2:1, v/v) as a negative control, and 10 µL of tetracycline HCl as a positive control were prepared. The Petri dish was placed in a refrigerator at a temperature of ± 5
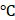
to effect the diffusion of the pre-electrospinning nanofiber solution and then incubated at 37
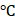
for 24 hours. The inhibition zone produced was then measured 21.

##### Microbial Penetration

It is necessary to determine the ability of nanofibers to hold microbes to figure out their suitability to be applied in wound dressings for diabetic wounds 14. The evaluation of microbial penetration was carried out according to Roy *et al*. (2010) with some modifications. It was performed by placing nanofibers measuring 1 × 1 cm over an agar medium in a Petri dish. Fifty microliters of a 10^7^ CFU/mL microbial culture were dropped on the nanofiber surface, followed by incubation at 37
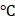
to simulate the body fluid temperature and 32
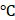
to simulate the skin surface temperature for 24 hours. The bacterial growth that formed was then visually observed 22.

#### *In Vivo* Diabetic Wound Healing Activity

Diabetic wound healing activity was examined using the methods by Lee *et al*. (2015), Fauzian *et al*. (2024), and Salami *et al*. (2021) with some modifications. All test procedures already received an ethical approval from the Ethical Committee of Institut Teknologi Bandung (No. KEHP/2025/271124BN/LDNR). The subjects on which the test procedures were applied were male Swiss Webster mice 8-10 weeks old and weighing 35-40 grams from CV Kencana Animal Breeding. At first, all mice were acclimatized for seven days at the laboratory and given standard pellets and drinking water *ad libitum*. The room used for the maintenance of the mice was maintained at 25-27 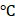
and 55-65% relative humidity and with a 12-hour illumination cycle 14, 23, 24.

Before excision to produce diabetic wounds, all mice were diabetically induced with repeated intraperitoneal injections of streptozotocin (STZ) dissolved in citrate buffer (50 mM, pH 4.5) at a dose of 40 mg/kg body weight for five days. The drinking water of the mice were substituted with 10% sucrose solution ad libitum for five days. The blood glucose level of each mouse was examined 14 days after the diabetes induction with a glucose meter (Family Dr.®, Allmedicus Co., Ltd., Gyeonggi-do, Republic of Korea). Only mice with glucose levels above 200 mg/dl and under 400 mg/dl were chosen and subjected to the next testing stage.

Mice indicated as diabetic were shaved at the posterior region with a shaving area of 6 × 3 cm^2^. The mice were given an intraperitoneal anesthetic injection of urethane at a dose of 1,300 mg/kg body weight. The anesthetized mice were given two wounds with a biopsy puncher down to the hypodermis layer. The wounds were then given six manners of treatment once a day. Mice number 1 until 4 received NFER on one wound and NF on the contralateral wound; mice number 5 until 8 received 10% rosemary ethanolic extract dispersed in vaseline album and vaseline album on the contralateral wound; and mice number 9 until 12 received 10% povidone iodine on one wound and 0.9% sodium chloride on contralateral wound. Each group consisted of four mice. The degree of wound healing was calculated using the equation below.



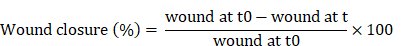



Wound closure was also calculated following the area under curve (AUC) method.



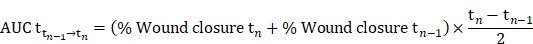



### Histological Analysis

Histological analysis was performed according to Fauzian *et al*. (2024). Briefly, the mice were euthanized on the last day of examination. Then, the tissue from the healed wound area was isolated in full thickness. The tissue sample was sectioned along the widest length of wound, fixed with a neutral paraformaldehyde buffer, dehydrated with a series of ethanol, cleaned with xylene, and embedded in liquid paraffin. A microtome was obtained from the biopsy, which was then stained with a hematoxylin and eosin (H&E) stain. Each slide was examined under an Olympus FluoView FV300 Confocal Laser Scanning Microscope (Olympus Corporation, Tokyo, Japan) equipped with a digital camera 24.

### Statistical Analysis

Statistical analyses were performed using GraphPad Prism version 10.2.3 (Dotmatics, Boston, MA, USA). Data are presented as mean ± standard deviation (SD). The antioxidant and antibacterial activities were analyzed using a **two-tailed unpaired Student's** ***t*****-test.** Each mouse received two wounds with paired treatment allocation, wound closure data for each paired treatment comparison were analyzed using a two-way mixed-effects model (REML), with treatment and time as fixed effects and mouse as the matched subject to account for repeated measurements over time and within-animal correlation. Multiple comparisons between treatments at each time point were performed using Tukey multiple-comparisons test. Significance was defined as *p-value* < 0.05.

## Results

### Phytochemical Screening of Rosemary Extract

Phytochemical screening of the rosemary leaf ethanolic extract indicated a rich profile of secondary metabolites, notably flavonoids, tannins, steroids, terpenoids, alkaloids, and coumarins. The presence of flavonoids, which are well known for their antioxidant, anti-inflammatory, and antimicrobial properties, suggests a potential role in mitigating oxidative stress within the wound microenvironment. Tannins, characterized by their astringent and antimicrobial activities, may contribute to enhanced wound contraction and reduced microbial colonization. Steroids and terpenoids, frequently associated with anti-inflammatory and tissue-regenerative effects, further support the extract's applicability in modulating inflammatory responses during wound healing. Additionally, alkaloids and coumarins, both recognized for their diverse bioactivities—including antimicrobial, vasodilatory, and antioxidant effects—provide further therapeutic value. Collectively, this diverse phytochemical composition underscores the multifaceted pharmacological potential of the rosemary extract and supports its incorporation into biomaterial systems aimed at promoting wound repair and tissue regeneration.

### Physical Characterization of Nanofiber

Macroscopically, the NFER displayed a uniform greenish coloration, consistent with the incorporation of rosemary-leaf ethanolic extract, and exhibited a smooth, continuous surface without visible defects such as bead formation or incomplete solvent evaporation. The fabricated nanofiber mat demonstrated structural uniformity, with a measured thickness of 102 ± 23.37 µm, indicating consistent deposition during the electrospinning process.

The morphology of the nanofibers was examined using scanning electron microscopy (SEM) at an accelerating voltage of 15 kV. SEM analysis revealed that both NFER and NF exhibited smooth, continuous fibers without bead formation or structural irregularities, suggesting effective electrospinning parameters and successful encapsulation and dispersion of the rosemary ethanolic extract within the polymer matrix 15.

The wettability of NFER was assessed using a CAAI 2320 contact angle goniometer, and the temporal changes in the water-surface contact angle are shown in Figure 4. At the initial measurement (0-10 s), the contact angle exceeded 90°, indicating that the NFER surface exhibited hydrophobic behavior.

The swelling behavior of NFER was determined gravimetrically using phosphate-buffered saline (PBS) supplemented with 0.5% Tween 20. NFER exhibited a swelling ratio of 481.09 ± 4.69%, whereas the unloaded NF showed a comparable swelling ratio of 433.87 ± 36.90%. The absence of a significant difference between the two samples is likely attributable to the intrinsic hydrophilicity of cellulose acetate, which dominates the water uptake profile regardless of extract incorporation. High swellability in nanofiber-based wound dressings is advantageous, as it supports the dissolution and diffusion of encapsulated bioactive compounds, thereby facilitating controlled release during wound treatment 25. Moreover, robust swelling capacity reflects the material's ability to efficiently absorb wound exudate, an essential requirement for maintaining moisture balance and promoting optimal wound-healing conditions 14.

### Encapsulation Efficiency

The encapsulation efficiency of the NFER was assessed using a modified version of the method described by Suwantong *et al*. (2008). The calculated encapsulation efficiency for NFER was 86.17 ± 3.02%. This result exceeded a previously reported encapsulation efficiency value of 75.4 ± 4.6% by Suwantong *et al*. (2008) for *Centella asiatica* extract encapsulated using the same polymer-solvent system 15. The higher efficiency achieved in this study (86.17%) suggests an improved incorporation of the rosemary extract into the cellulose acetate nanofiber matrix compared to the *Centella asiatica* extract. This indicates a successful and efficient encapsulation process for the rosemary extract formulation.

### *In Vitro* Release

The cumulative release profile of rosmarinic acid from NFER is shown in Figure 5. The release behavior exhibited a biphasic pattern, initially characterized by an immediate release within the first four hours—reaching 38.85 ± 0.02%—followed by a slower, sustained release phase extending to six hours. This rapid early release is typical of nanofibers with high porosity, where surface-localized or weakly bound compounds diffuse readily into the medium 26. Although sustained release may be further optimized using alternative fabrication strategies such as coaxial electrospinning 27, the observed burst release can be advantageous in wound treatment by providing an immediate therapeutic effect, followed by a more controlled and prolonged delivery of the active compound 28.

### Antioxidant Activity

During the inflammatory phase of normal wound healing, platelet-derived growth factor (PDGF) recruits neutrophils and monocytes, which function to eliminate invading microorganisms and secrete mediators that promote subsequent repair processes. Neutrophils, in particular, generate proteolytic enzymes and reactive oxygen species (ROS) to kill pathogens. In diabetic wounds, however, hyperglycemia and the accumulation of advanced glycation end-products (AGEs) disrupt redox homeostasis, leading to excessive and prolonged ROS production and, consequently, an extended inflammatory phase that impairs healing 29. In this study, the antioxidant activity of NF and NFER was evaluated using the DPPH assay, a widely employed method due to the stability of the DPPH radical. The assay relies on the reduction of DPPH, which contains an unpaired electron on its nitrogen atom, through hydrogen donation by antioxidant compounds, resulting in the formation of the reduced species DPPH•-H 30. Antioxidant capacity was expressed as both ascorbic acid equivalent (Figure 6a) and rosemary extract equivalent (Figure 6b).

### Antibacterial Activity

Diabetic wounds exhibit a heightened vulnerability to bacterial colonization, primarily as a consequence of compromised immune function and impaired tissue repair mechanisms. Among the microorganisms commonly associated with these chronic wounds, *Staphylococcus aureus* and* Staphylococcus epidermidis* are particularly prevalent, often occurring at significantly elevated levels relative to those found in non-diabetic or healthy wounds. As shown in **Table 1**, the antibacterial efficacy of NFER demonstrated markedly stronger inhibitory activity than rosemary extract across the tested bacterial strains. Notably, in several assessments, NFER exhibited antibacterial performance comparable to—or surpassing—that of the reference antibiotic, Tetracycline HCl, underscoring its potential as a promising alternative or complementary therapeutic agent in wound management.

### Microbial Penetration

Nanofiber-based wound dressings should mimic the structural and functional characteristics of the extracellular matrix to shield diabetic wounds from external microbial contamination and thereby promote accelerated healing 22, 31. In this study, microbial penetration testing was performed using *Staphylococcus aureus* and *Staphylococcus epidermidis* incubated at 32 °C and 37 °C for 24 hours. Figure 7 illustrates the agar plates before and after removal of the NF and NFER mats that had been placed over bacterial suspensions.

### Diabetic Wound Healing *In Vivo*

The diabetic wound-healing efficacy of NFER was assessed using a full-thickness excision wound model in streptozotocin-induced diabetic male Swiss Webster mice. According to Figure 8, both NFER and NF promoted wound healing throughout the 14-day treatment period. In addition, as shown in Figure 9, NFER achieved an area under the wound-closure curve of 97.68 ± 3.72%, and NF achieved 94.72 ± 1.39%, both significantly higher (p < 0.05) than the 0.9% sodium chloride group (85.32 ± 3.31%) or 10% Povidone iodine (86.20 ± 5.66%). However, the wound-healing outcomes of NFER and NF were comparable, indicating that the present *in vivo* study couldn't conclusively distinguish the additional therapeutic contribution of rosemary extract from the beneficial effect of the nanofiber dressing itself.

Histopathological evaluation (Figure 10) qualitatively supported the wound-healing findings. Skin tissues treated with NFER, NF, ointment, or 10% povidone iodine showed no apparent neutrophil or lymphocyte infiltration. In contrast, tissues treated with ointment base or 0.9% sodium chloride exhibited lymphocytic infiltration indicative of chronic inflammation. Representative H&E-stained sections also suggested that NFER treatment has thicker epidermal thickness than all other groups, demonstrating promoted wound healing and supporting its superior therapeutic potential 32. However, because histological assessment was qualitative and based on representative images without blinded quantitative analysis, these observations should be interpreted as supportive rather than definitive of improved wound healing.

## Discussion

### Phytochemical Screening

Flavonoids and tannins identified in the rosemary leaf ethanolic extract exhibit pronounced antioxidant capacity capable of neutralizing reactive oxygen species (ROS), thereby mitigating oxidative stress—a major contributor to delayed and dysfunctional healing in diabetic wounds. Beyond their antioxidative action, these polyphenolic compounds have been shown to modulate cellular proliferation, enhance cytoprotective pathways, and stabilize extracellular matrix components, collectively supporting tissue repair processes 33. Terpenoids, steroids, and alkaloids within the extract further contribute to its therapeutic profile through documented broad-spectrum antimicrobial activities, which aid in suppressing pathogenic colonization and maintaining a more favorable wound microenvironment 34. Moreover, several phytochemicals, particularly flavonoids and terpenoids, are associated with anti-inflammatory and antidiabetic mechanisms, including inhibition of pro-inflammatory cytokines, regulation of NF-κB-mediated pathways, and improvement of glucose homeostasis—actions that are particularly relevant in reducing chronic inflammation characteristic of diabetic wounds 35. Coumarins provide additional synergistic benefits due to their antioxidant, anti-inflammatory, and vasomodulatory properties, which can enhance microcirculation and support granulation tissue formation. Collectively, this diverse and functionally complementary phytochemical composition underscores the multifunctional bioactivity of rosemary extract and strengthens its potential application as a natural therapeutic agent for improving wound repair in diabetic conditions.

### Physical Characterization of Nanofiber

Fiber diameters were quantified using ImageJ software by measuring 50 randomly selected points per sample. The average diameter of NFER was 237.51 ± 39.37 nm, whereas the unloaded NF displayed a smaller diameter of 200.40 ± 39.37 nm. The observed increase in fiber diameter following incorporation of the extract is consistent with the expected rise in solution viscosity and the concurrent reduction in electrical conductivity prior to electrospinning, both of which influence jet stretching and fiber thinning during the spinning process 36. These results indicate that the presence of rosemary extract modulated the physicochemical properties of the precursor solution, leading to the formation of slightly thicker but well-defined nanofibers.

According to wettability property of NFER, such transient hydrophobicity is commonly observed in cellulose acetate-based nanofibers and can influence the early wound interface by limiting rapid fluid spreading. From the previous studies, hydrophobic surfaces have also been reported to impede bacterial adhesion and biofilm formation for both Gram-positive and Gram-negative species, thereby contributing to infection control at the wound site 37, 38. By 30 s, however, the contact angle decreased markedly, reflecting a transition toward a hydrophilic surface. This shift is advantageous for wound management, as hydrophilicity promotes efficient absorption of wound exudate and supports moisture balance conducive to tissue regeneration 14, 39. Together, these results suggest that NFER exhibits time-dependent wettability, which may contribute to early antimicrobial protection according to previous reports.

High swelling capacity in nanofiber-based wound dressings is a critical functional property, as it enhances the dissolution and subsequent diffusion of encapsulated therapeutic agents, thereby enabling a more controlled and sustained release profile during wound management 32. This characteristic is particularly important for bioactive compound delivery, where regulated release kinetics can significantly improve antimicrobial, anti-inflammatory, or regenerative outcomes. Furthermore, a pronounced ability to swell indicates the material's effectiveness in absorbing wound exudate, which is essential for maintaining an optimal moist wound environment 14. Adequate exudate management not only prevents maceration of the surrounding tissue but also supports cellular proliferation, migration, and extracellular matrix formation—key processes required for efficient and accelerated wound healing.

### *In Vitro* Release

Kinetic modeling of the release behavior of rosmarinic acid from the NFER system revealed that the experimental data aligned with several widely applied kinetic models, including zero-order, first-order, Higuchi, and Korsmeyer-Peppas. Comparative evaluation of model fitting parameters showed that the Korsmeyer-Peppas model exhibited the highest correlation coefficient (R² = 0.9644), indicating superior predictive accuracy relative to the other models. This strong correlation suggests that the release process is governed predominantly by anomalous (non-Fickian) transport, in which both diffusion and polymer matrix relaxation contribute to the overall release mechanism. The applicability of the Korsmeyer-Peppas model further implies that the nanofiber structure and polymer-drug interactions play critical roles in modulating the release rate of rosmarinic acid. Collectively, these findings highlight the mechanistic complexity of the release system and demonstrate that the Korsmeyer-Peppas model provides the most robust description of rosamarinic acid liberation from NFER.

The Korsmeyer-Peppas kinetic model provides a widely applicable framework for characterizing drug release behavior from polymeric delivery systems, including electrospun nanofibers. This model enables the estimation of drug release at a given time and offers mechanistic insight through the release exponent “n,” which reflects the dominant transport pathway. In the present study, the calculated n value was 0.6948, indicating that rosmarinic acid release from NFER followed a non-Fickian, or anomalous, transport mechanism. This type of release behavior suggests that drug diffusion and polymer relaxation (swelling) occur at comparable rates, with neither process acting as the sole controlling factor. Such anomalous release is typically associated with gradual polymer swelling and chain rearrangement, which occur concurrently with the diffusion of the encapsulated compound, resulting in a combined and balanced contribution from both mechanisms.

### Antioxidant Activity

One gram of NFER yielded 2.067 ± 0.004 mg ascorbic acid equivalents, a value markedly higher than that obtained for the NF formulation (0.154 ± 0.000 mg). This enhancement is likely attributable to the incorporation of rosemary extract, which demonstrated notable radical-scavenging activity with an IC₅₀ of 216.69 ± 1.01 mg/L. Furthermore, one gram of NFER corresponded to 147.81 ± 0.282 mg of rosemary extract, whereas NF contained only 4.88 ± 0.000 mg, underscoring the substantially greater phytochemical loading achieved in the enriched formulation.

The pronounced antioxidant capacity of rosemary extract is well-documented and is primarily associated with its high phenolic content, particularly carnosic acid, carnosol, and rosmarinic acid 40, in addition to flavonoids and tannins identified through phytochemical screening. Recent findings by Al-Jaafreh (2024) further support these observations, showing that ethanolic rosemary extract exhibits significantly higher total phenolic and flavonoid contents than aqueous or methanolic extracts, a trend that correlates strongly with enhanced antioxidant efficacy 41.

### Antibacterial Activity

Diabetic wounds are also prone to bacterial colonization due to impaired immune function, with *Staphylococcus aureus* and *Staphylococcus epidermidis* frequently identified at elevated levels compared with healthy wounds. These bacteria contribute to delayed healing by producing proteolytic factors that compromise the skin barrier 42, while *S. aureus* additionally disrupts neutrophil extravasation and impairs phagocytosis 43. Consequently, antibacterial interventions are essential for effective diabetic wound management. Rosemary ethanolic extract has documented antibacterial properties 44, which were evaluated in this study using the well diffusion method for NFER and NF solutions, and the disc diffusion method for pure rosemary extract and the acetone-DMAc (2:1, v/v) solvent system. Variations in inhibition zones among the samples were likely due to differences in viscosity and the presence of cellulose acetate in NFER, which slows drug release into the agar matrix 45. Antibacterial activity was evaluated against *S. aureus* and *S. epidermidis*, both clinically relevant pathogens in chronic diabetic wounds.

### Microbial Penetration

According to Figure 7, both NF and NFER completely prevented bacterial penetration under all evaluated conditions, indicating their effectiveness as physical barriers analogous to the native extracellular matrix. This barrier function may be partially associated with the initially hydrophobic surface of the NF and NFER mats as indicated by the water contact angle measurements. Previous studies have reported that the surface hydrophobicity may influence bacterial adhesion, particulary for microorganisms with relatively hydrophobic cell surface, such as *S. aureus* 46. However, bacterial adhesion was not directly evaluated in the present study' therefore the contribution of surface hydrophobicity to the observed barrier function remains a proposed mechanism rather than a directly demonstrated finding. In addition, the dense and interconnected fiber networks, collectively enhance the materials' ability to restrict microbial passage, thereby reinforcing their protective role as advanced wound-dressing systems.

### Diabetic Wound Healing *In Vivo*

Figure 8 and 9 illustrates that NFER markedly enhanced wound-closure rates compared with physiologic sodium chloride, with statistically significant differences observed on Days 2, 10, and 14 (p < 0.05). During the first two hours, wound closure reached only 30% with 0.9% sodium chloride treatment, whereas NFER treatment achieved approximately 55%. The percentage of wound closure following treatment with sodium chloride reached only about 85% (days 0-14). In contrast, with NFER treatment, wound closure reached 98% in period of 14 days. The superior wound-healing performance of NFER is likely attributable to the synergistic activity of the bioactive phytochemical constituents present in the rosemary ethanolic extract. As reported by Alizargar *et al*. (2012), these constituents can promote diabetic wound repair by stimulating fibroblast proliferation, enhancing collagen deposition, and accelerating granulation tissue formation—all critical processes often impaired under hyperglycemic conditions. Additionally, rosemary-derived compounds have been shown to inhibit collagenase activity and reduce bacterial burden, thereby preserving newly formed extracellular matrix and supporting a more favorable healing environment 47. The current findings align with those of Elbhnsawi *et al*. (2023), who documented similar improvements in wound-closure kinetics in animal models infected with *Staphylococcus aureus*, further reinforcing the antimicrobial and tissue-regenerative potential of rosemary-based formulations 48. In accordance with standard diabetic wound-care recommendations, which emphasize consistent cleansing and the use of simple non-active dressings, 0.9% sodium chloride applied with hydrophilic gauze served as the negative control in this study, providing a clinically relevant baseline for comparison 49, 50. In the fact, numerous studies demonstrate an increase in the pharmacological activity of extracts after they are formulated as nanoparticles 51, 52.

One of the limitations of this study is that the antibacterial activity was evaluated using the pre-electrospinning formulation rather than the intact electrospun nanofiber dressing or its released contents. Consequently, although the antibacterial properties of the incorporated rosemary extract were demonstrated, the present study does not directly confirm antibacterial activity of the finished dressing after electrospinning. Future studies should evaluate antibacterial efficacy using intact nanofiber dressings or released constituents under conditions that better simulate clinical application.

Another limitation of this study is that the *in vivo* wound healing outcomes of rosemary extract-loaded (NFER) and unloaded (NF) nanofiber dressings were comparable. The area under the wound-closure curves for NFER and NF did not differ significantly, indicating that the present study could not conclusively distinguish the additional therapeutic contribution of rosemary ethanolic extract from the beneficial of nanofiber dressing itself. The absence of a pronounced difference may reflect the limited amount of extract released from the nanofibers at equilibrium (0.002 mg of rosmarinic acid, equivalent to 0.3 mg of ER after 240 minutes, based on the typical rosmarinic acid content of 1.33-12.40 mg per gram of rosemary) 53. Moreover, effective diabetic wound therapies require sustained moisture retention, bacterial inhibition, exudate regulation, and oxygen permeability 54. In the present study, the microbial penetration test confirmed that both NF and NFER effectively inhibited the passage of Staphylococcus aureus and Staphylococcus epidermidis, supporting their suitability as wound dressings. Additionally, NF and NFER exhibited comparable swelling behaviour, further indicating their ability to regulate wound moisture.

Therefore, while NFER demonstrated favourable wound-healing performance, the present findings do not allow the therapeutic effect of rosemary extract to be clearly separated from that of the nanofiber dressing. Further studies involving optimized extract loading, prolonged release profiles, and mechanistic investigations are warranted to clarify the specific contribution of rosemary extract to diabetic wound healing.

Chronic diabetic wounds represent a multifactorial pathological condition characterized by impaired healing processes. These wounds typically fail to progress through normal reparative phases due to elevated reactive oxygen species (ROS), bacterial colonization, and excessive exudate production 42, 55. Rosemary ethanol extract has been widely reported to possess antioxidant and antibacterial properties, and its incorporation into nanofiber formulations has demonstrated potential to enhance diabetic wound healing outcomes 22, 40, 44. Although *in vivo* wound closure assessments have provided limited evidence of significant therapeutic improvement, *in vitro* evaluations offer more compelling data. Antioxidant assays, particularly those employing the DPPH method, indicate that nanofiber-encapsulated rosemary ethanol extract (NFER) exhibits substantially stronger antioxidant capacity—expressed as rosemary extract equivalence and ascorbic acid equivalence—compared to nanofibers without extract (NF). ROS play a critical regulatory role in the inflammatory phase of wound healing by supporting antimicrobial defence mechanisms. However, ROS levels are markedly elevated in individuals with diabetes, driven by hyperglycaemia and the accumulation of advanced glycation end-products (AGEs) 56. Excessive ROS disrupt redox homeostasis across the inflammatory, proliferative, and remodelling phases, thereby prolonging wound healing. Antioxidants are therefore essential for restoring physiological ROS balance and promoting tissue repair 29. Antibacterial assays further demonstrate that NFER exhibits significant inhibitory activity against *Staphylococcus aureus* and *Staphylococcus epidermidis*, surpassing that of 10% ethanol rosemary extract (ER). Diabetic wounds are particularly susceptible to infection, often involving increased microbial burden and greater bacterial diversity, with *S. aureus* and *S. epidermidis* frequently implicated 40, 57. Consequently, antimicrobial activity is a critical determinant of effective wound healing. In addition to its antioxidant and antimicrobial functions, NFER supports wound repair by enhancing exudate absorption and providing a physical barrier against bacterial contamination from the external environment 14. Collectively, these properties highlight the potential of NFER as a multifunctional adjunct in the management of chronic diabetic wounds.

Nanofibers represent an efficient drug-delivery platform due to their small fiber diameter and correspondingly high surface area, which enable enhanced interaction with the wound environment. When formulated using biocompatible polymers, nanofibers can facilitate controlled drug release while exhibiting improved antibacterial and antioxidant properties, thereby supporting diabetic wound healing 58. Numerous studies have demonstrated the therapeutic potential of plant-extract-loaded nanofibers. Islam *et al*. (2022) developed polyvinyl alcohol (PVA)/chitosan nanofibers incorporating ethanol extract of *Malva sylvestris* flowers, which promoted diabetic wound repair through sustained release and antibacterial activity 59. Derakhshan *et al*. (2022) designed a trilayer nanofiber architecture comprising a hydrophobic polycaprolactone (PCL) outer layer for environmental protection, a PCL-collagen middle layer, and a wound-facing layer containing *Melilotus officinalis* extract, collectively supporting tissue regeneration in diabetic wounds 60. Similarly, Salami *et al*. (2021) reported that PCL-collagen nanofibers loaded with bitter melon fruit extract improved wound closure in a concentration-dependent manner, aided by the nanofiber barrier against external microbial intrusion 14. Almasia *et al*. (2021) produced polyurethane/Pluronic F127 nanofibers containing encapsulated plant extracts within cross-linked gelatin nanoparticles; these demonstrated superior wound healing compared with unloaded or non-encapsulated extract nanofibers, attributed to antibacterial activity against *Escherichia coli* and *Staphylococcus aureus* and the formation of thicker re-epithelialized tissue 61. Collectively, these findings highlight the capacity of nanofibers to deliver an initial burst of active compounds followed by sustained release, ensuring adequate and prolonged bioactive availability at the wound site.

## Conclusion

Nanofibers incorporated with rosemary ethanolic extract (NFER) were successfully fabricated, exhibiting smooth and continuous morphology with a characteristic green hue and an average fiber diameter of 237.51 ± 39.37 nm. The nanofibers displayed initial hydrophobic behavior within the first 10 seconds of water contact and demonstrated a high swelling capacity of 481.09 ± 4.69%. Rosemary extract was efficiently encapsulated within the nanofibers, with an encapsulation efficiency of 86.17 ± 3.02%. *In vitro* release profiling revealed that rosmarinic acid release from NFER followed the Korsmeyer-Peppas kinetic model, indicating an anomalous transport mechanism. Both NFER and NF effectively prevented bacterial penetration, demostrating that the nanofiber dressing itself serves as an effective physical barrier. In the diabetic wound model, NFER significantly enhanced wound-healing activity *in vivo* compared with the 0.9% sodium chloride control. However, because wound-healing outcomes were comparable between NFER and unloaded nanofibers, the present study could not conclusively distinguish the addition therapeutic contribution of rosemary extract from the beneficial effect of the nanofiber dressing itself. Overall, these findings highlight the potential of NFER as a multifunctional wound dressing, while further studies are required to clarify the specific contribution of rosemary extract to diabetic wound healing.

## Figures and Tables

**Figure 1:**
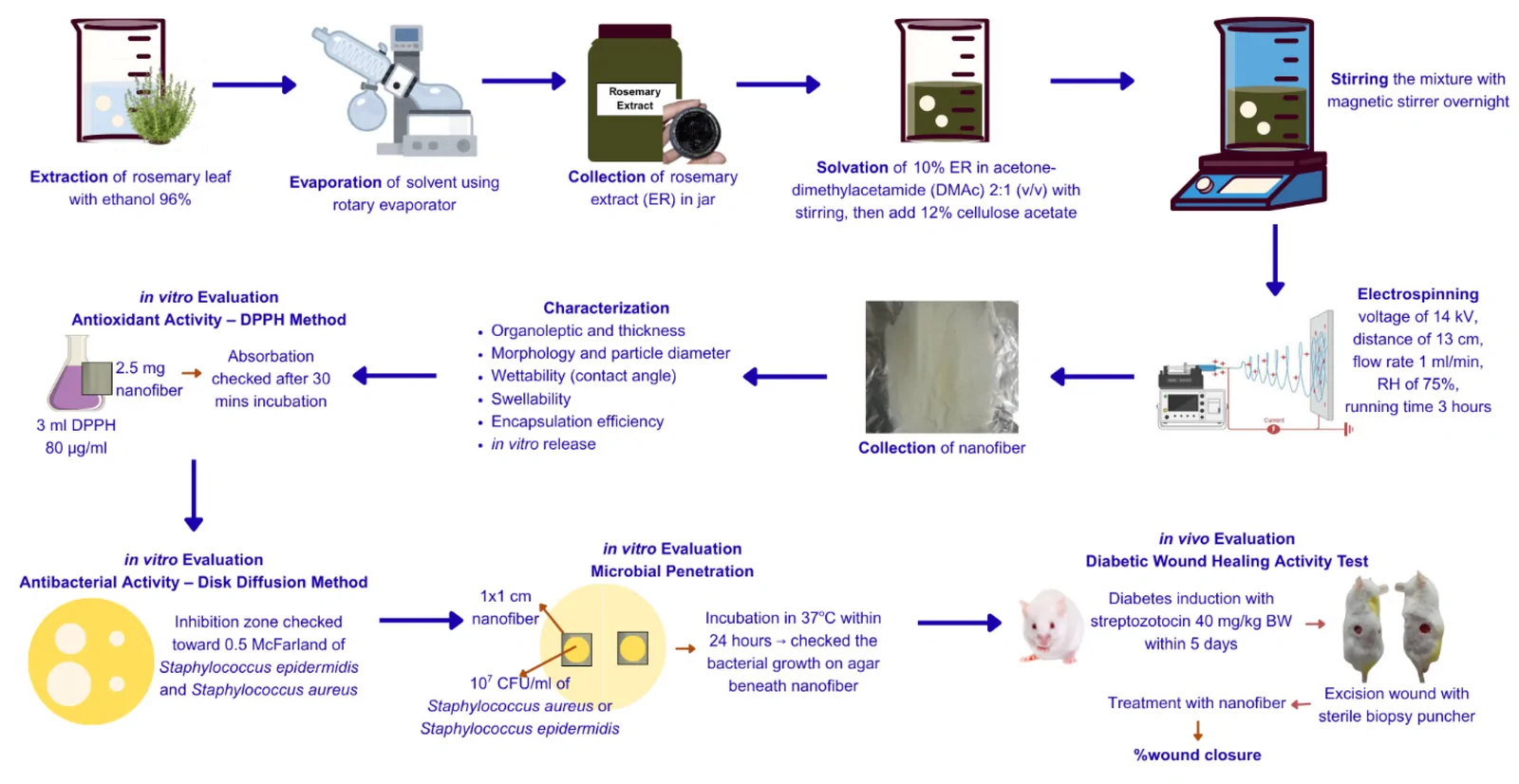
The schematic figure of this research.

**Figure 2:**
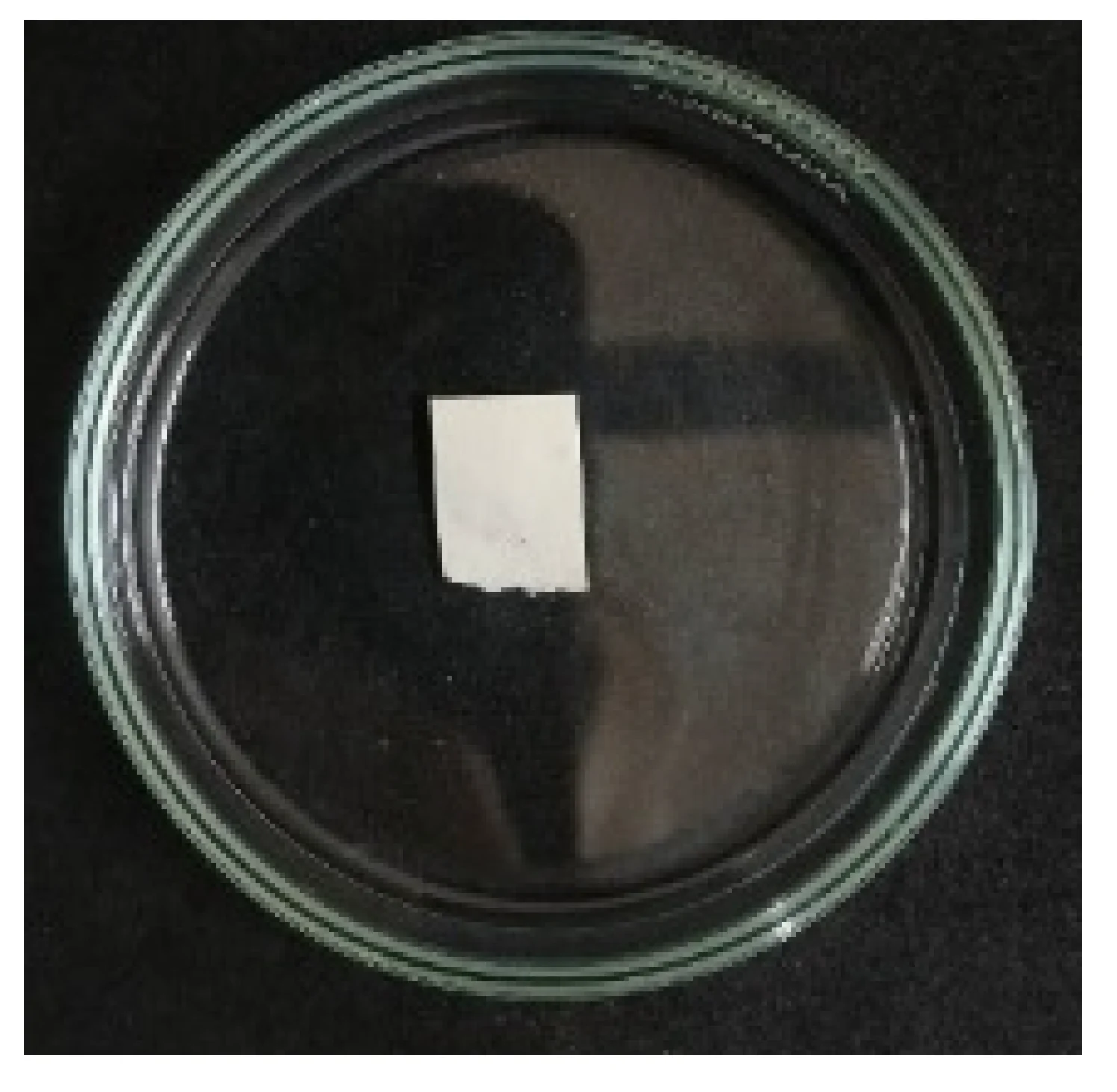
Visual of NFER.

**Figure 3:**
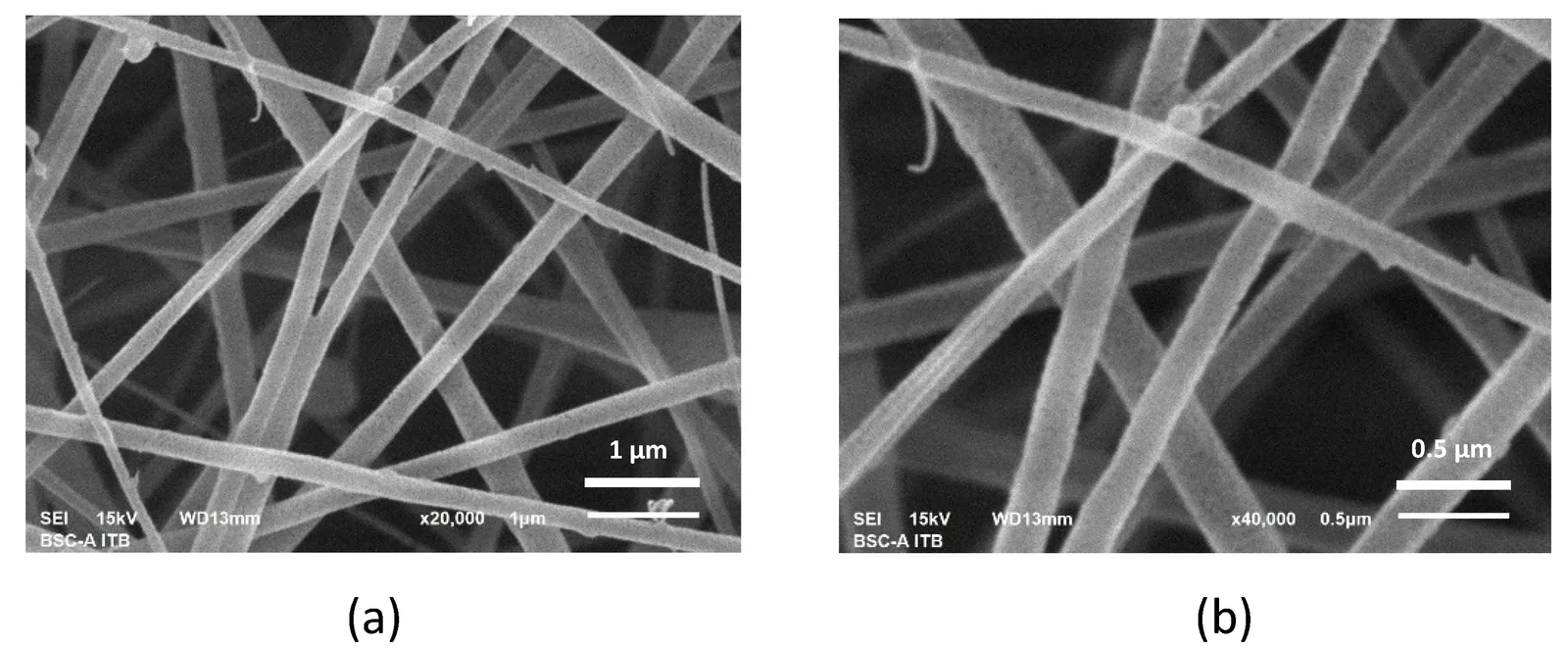
Microscopic of NFER with SEM, magnification (a) 20.000× and (b) 40.000×.

**Figure 4:**
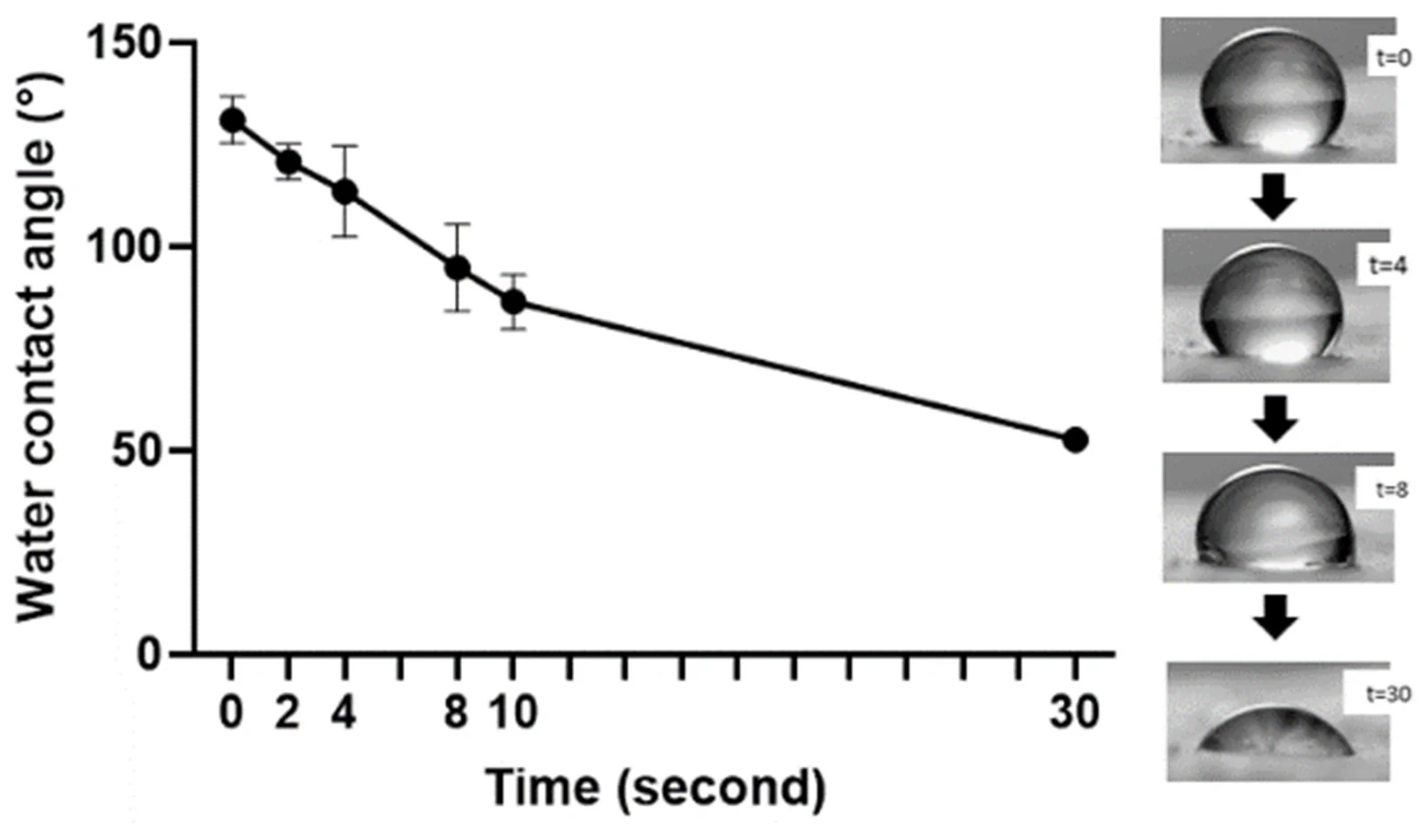
Water contact angle within NFER and water (left picture) and water visualization toward NFER within time (right picture). Data presented as mean ± deviation standard (n=3).

**Figure 5:**
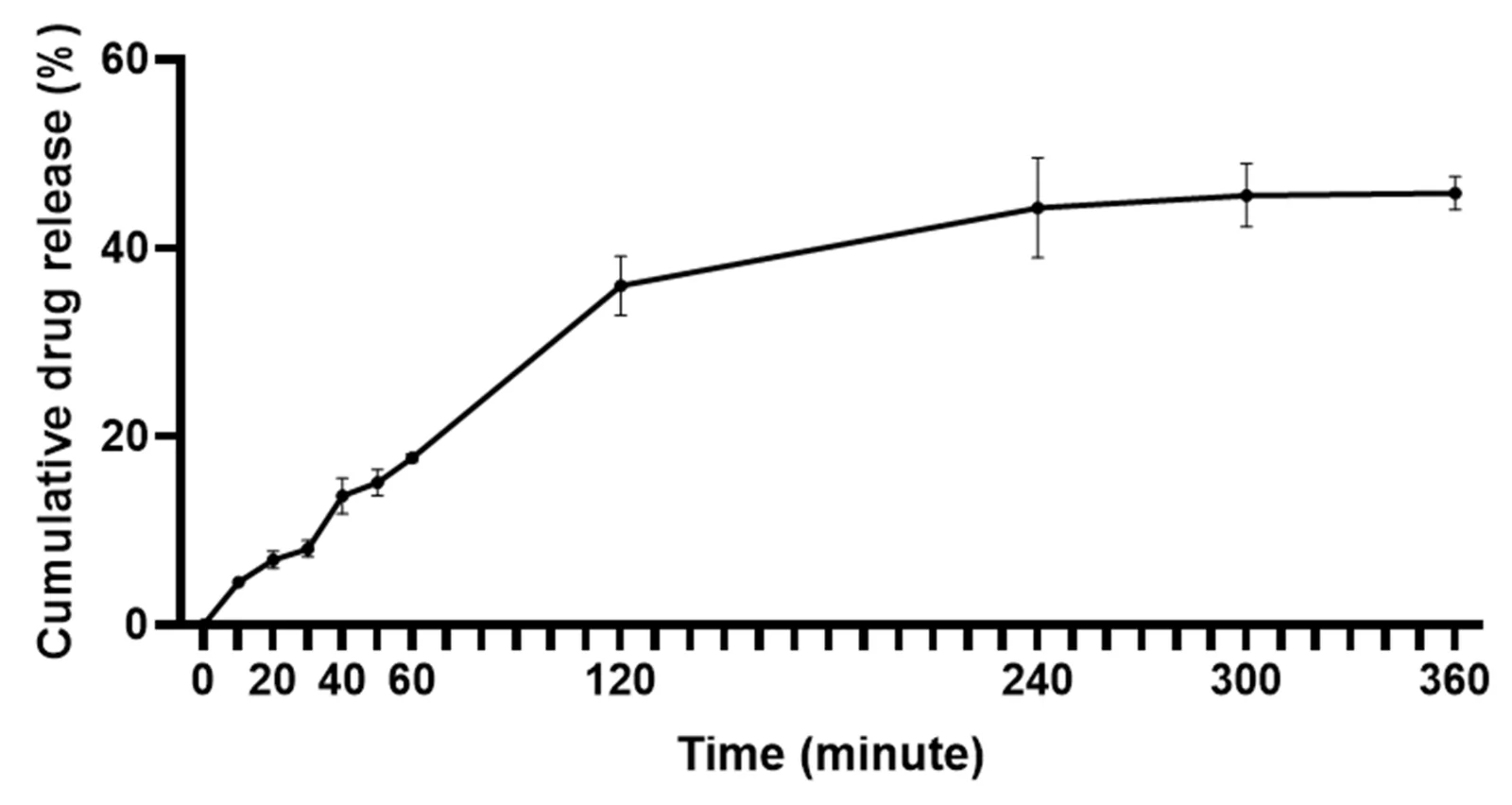
Release profile of rosmarinic acid from NFER *in vitro.* Data presented as mean ± deviation standard (n=3).

**Figure 6:**
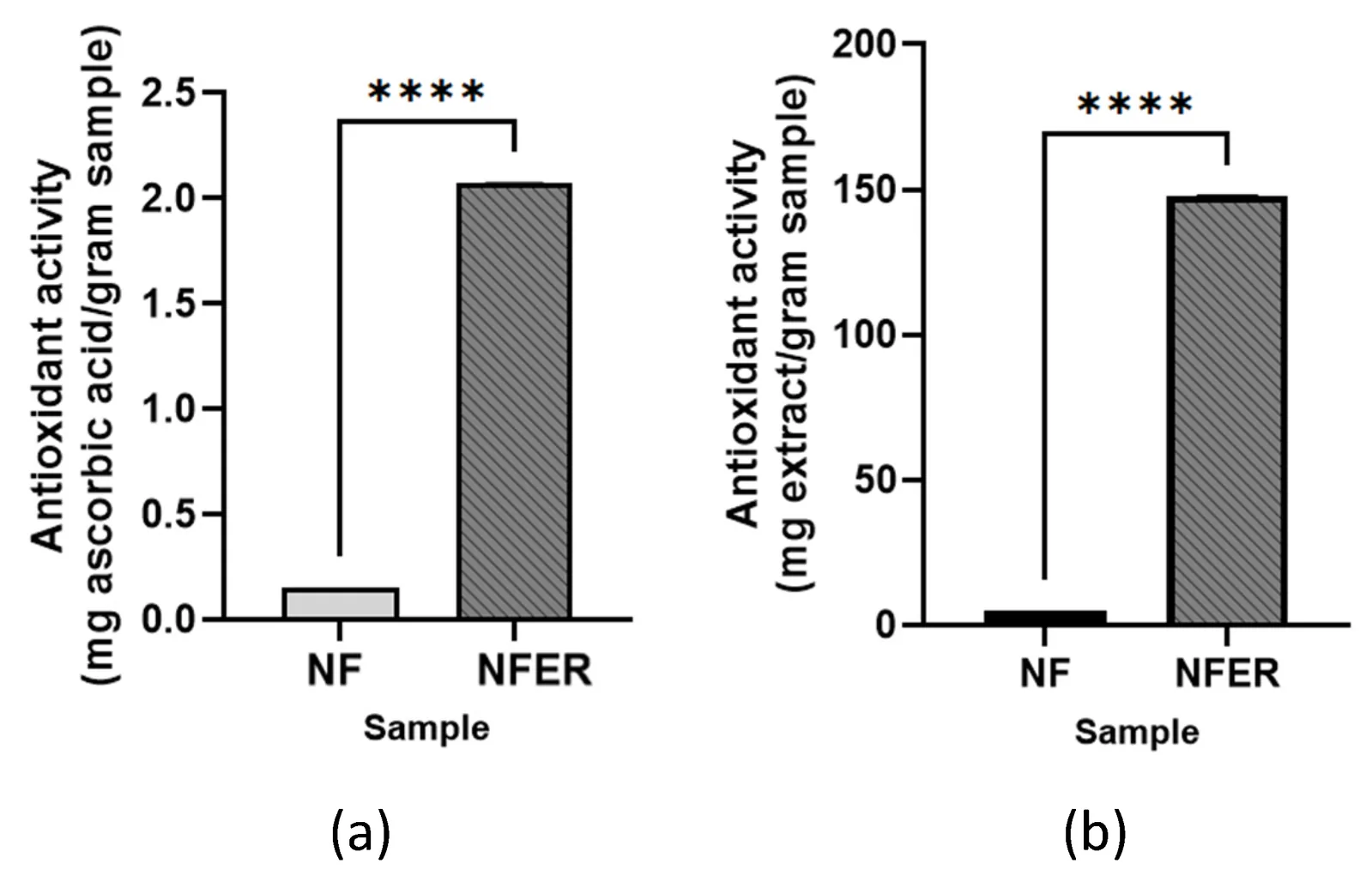
Antioxidant activity of NF and NFER in (a) ascorbic acid equivalent and (b) rosemary extract equival ent. Data declared as average ± deviation standard (n=3). ****Significantly different (*p-value*<0,0001).

**Figure 7:**
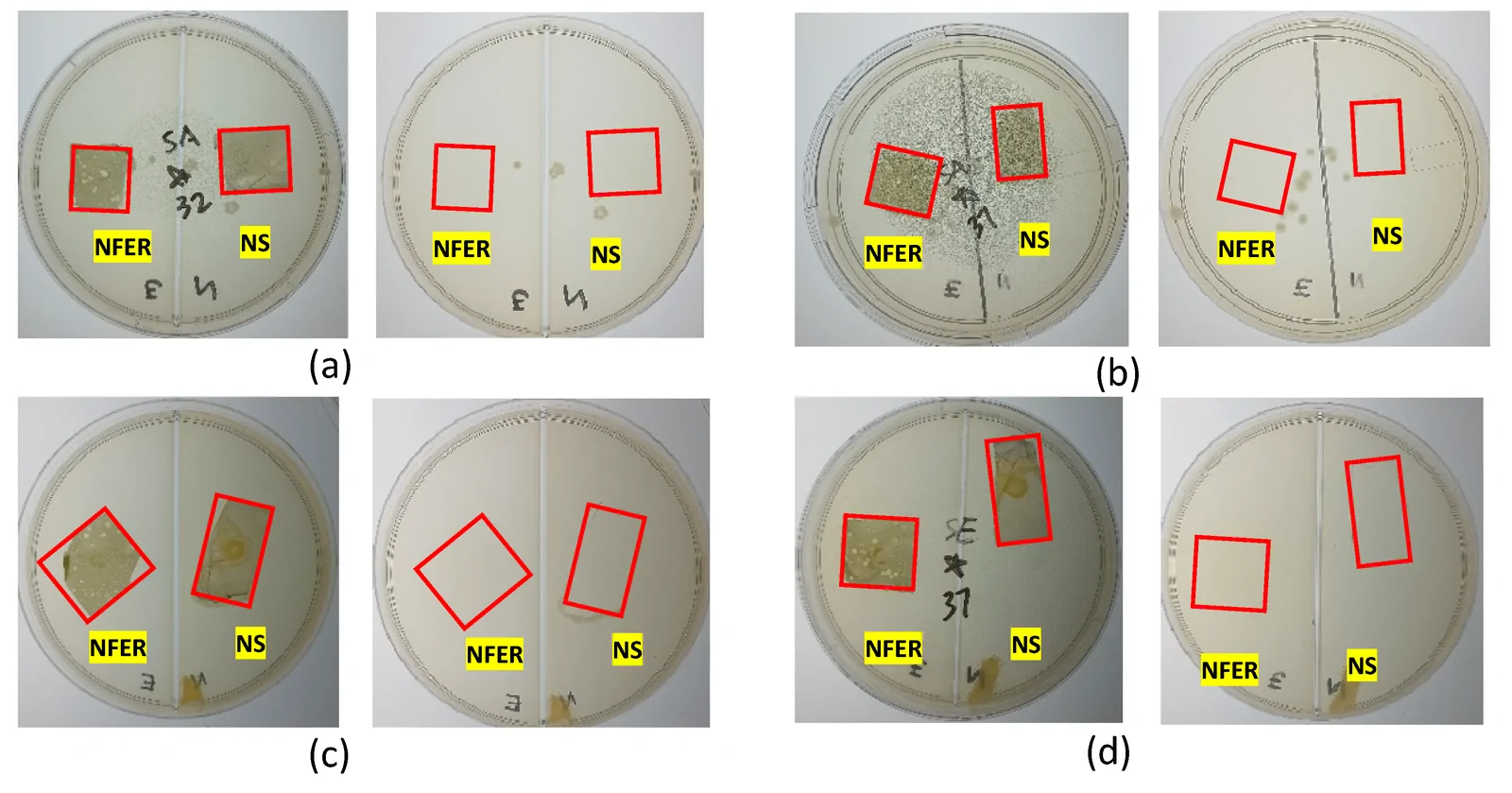
Microbial penetration, before (left picture) and after (right picture) (a) *Staphylococcus aureus* at 32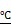, (b) *Staphylococcus aureus* at 37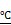, (c) *Staphylococcus epidermidis* at 32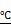, and (d) *Staphylococcus epidermidis* at 37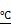.

**Figure 8:**
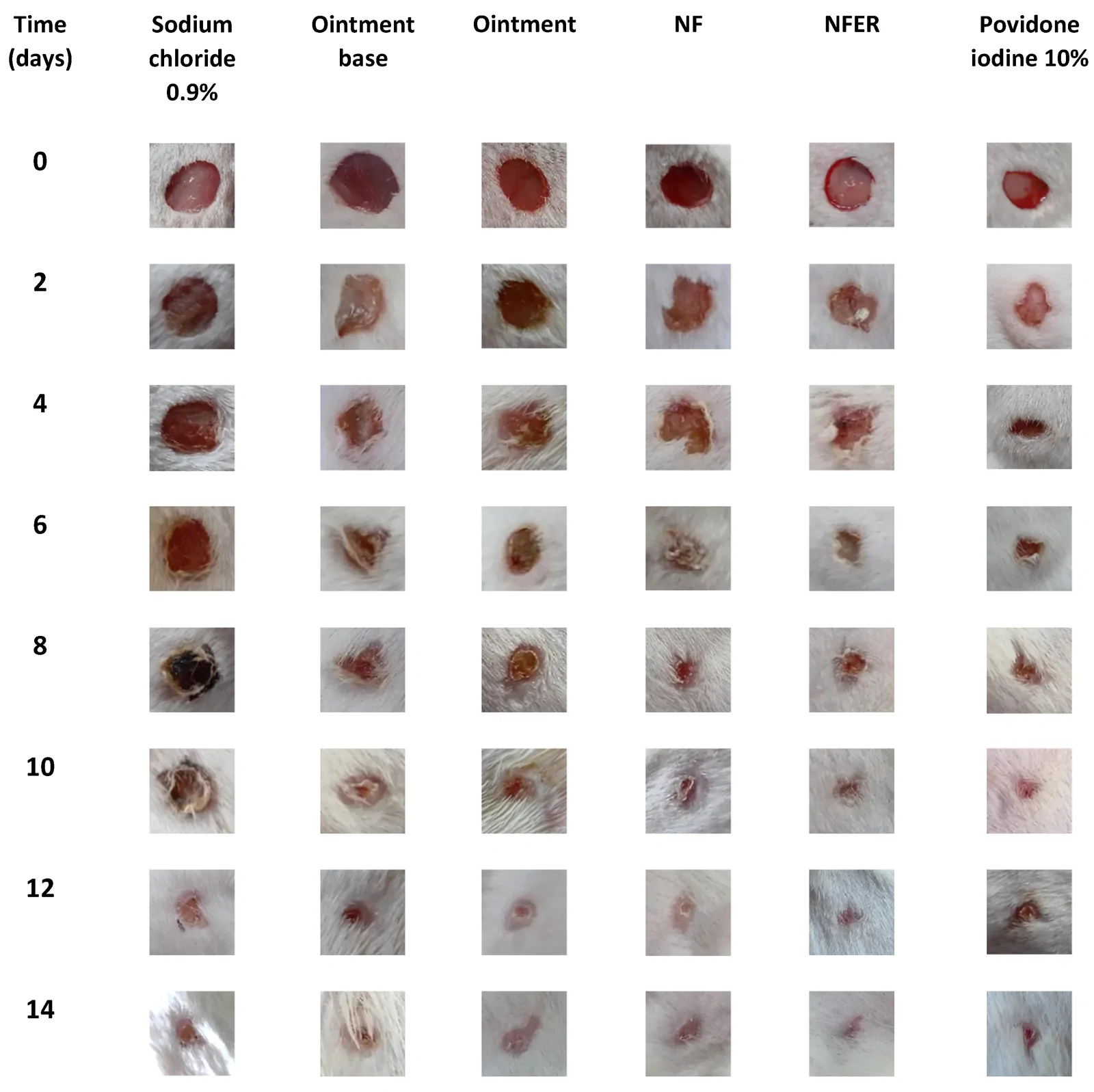
Diabetic wound healing *in vivo* test in period of 14 days after treated by several dosage forms.

**Figure 9:**
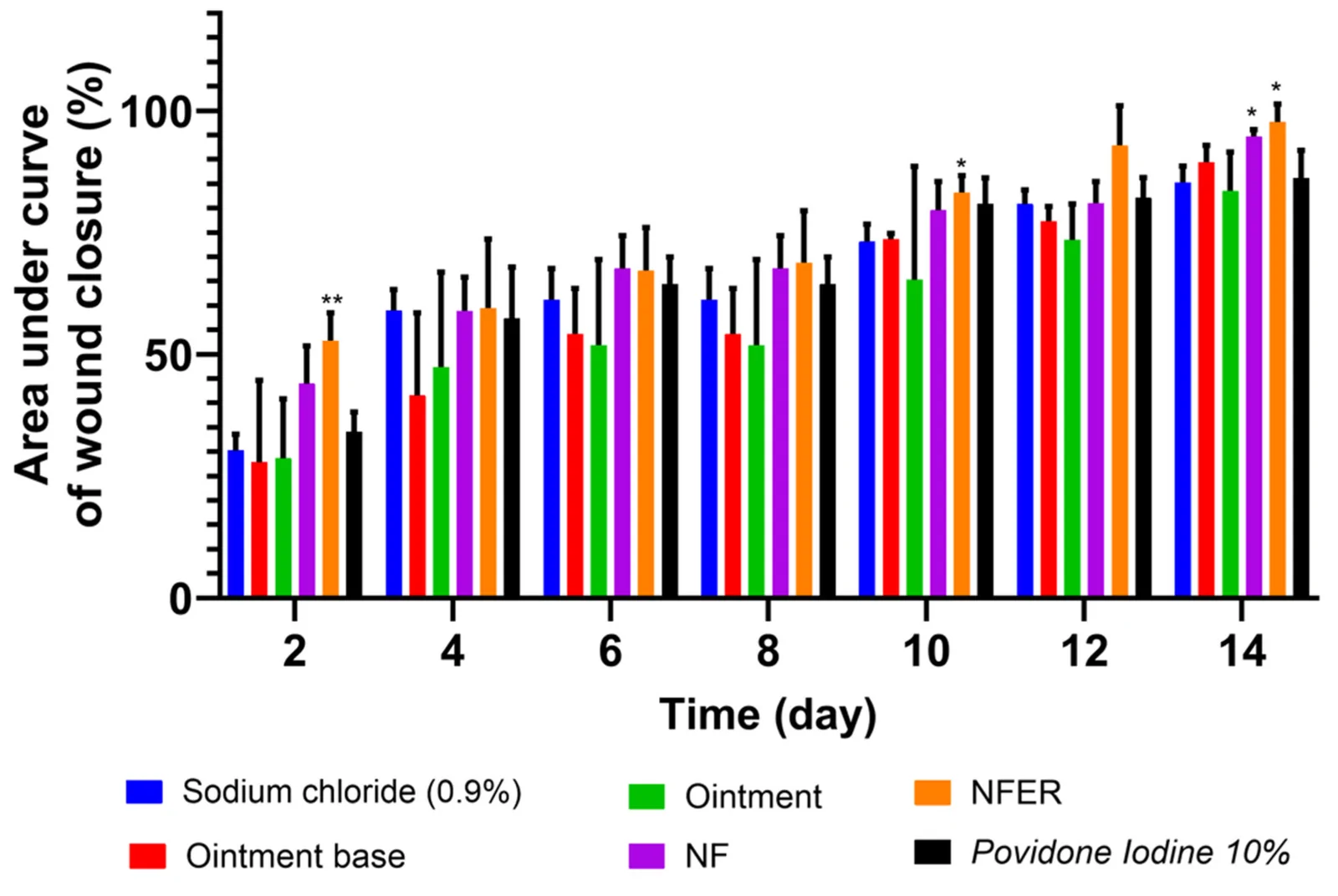
Diabetic wound healing activity. Data presented as mean ± deviation standard (n=4) *significantly different toward sodium chloride 0.9% (*p value*<0.05).

**Figure 10:**
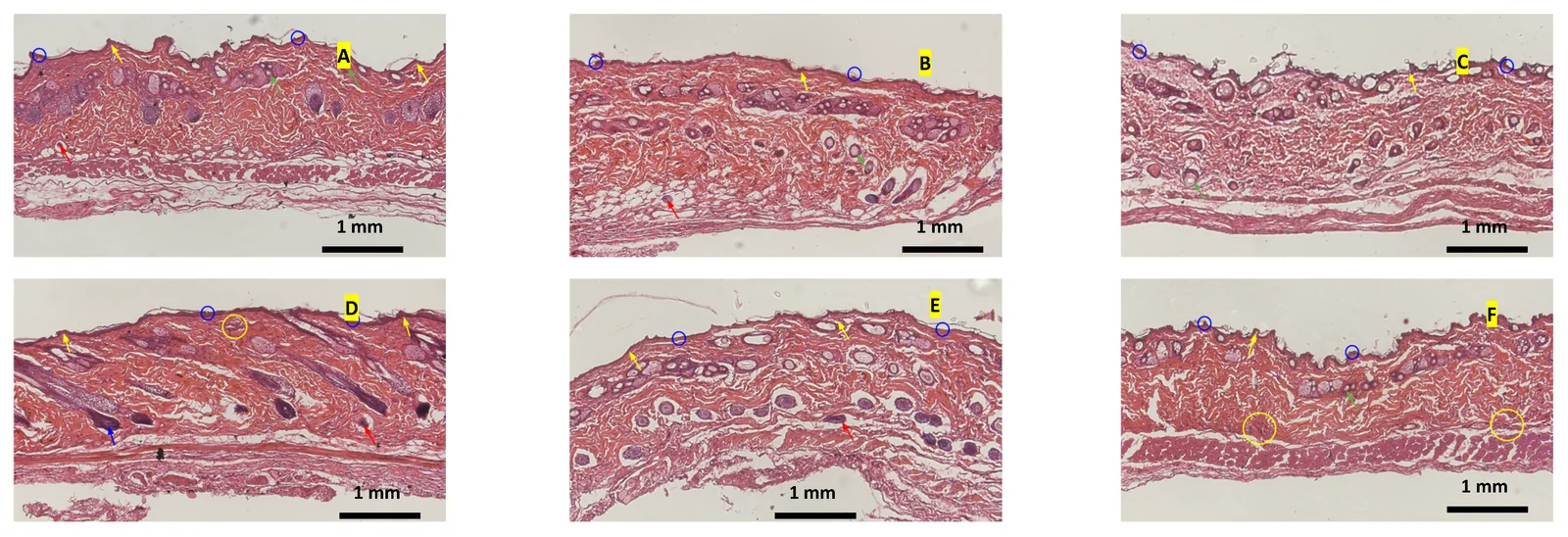
Histopathological analysis of skin (A) NFER, (B) NF, (C) Ointment, (D) Ointment Base, (E) 10% povidone iodine, (F) 0.9% sodium chloride. Stained with Hematoxylin-eosin (H&E). Visualization at 10 times magnification. Blue circles indicate epidermis, red arrows indicate blood vessels, yellow arrows indicate dermal papillae, green arrows indicate sebaceous glands, blue arrows indicate dermal appendages, yellow circles indicate necrotic tissue.

**Table 1:** Antibacterial Activity in Zone of Inhibition (mm)

Samples	Zone of Inhibition (mm)	
	*Staphylococcus aureus*	*Staphylococcus epidermidis*
Ac-DMAc (2:1, v/v)	0.00 ± 0.00	0.00 ± 0.00
Tetracycline HCl	11.50 ± 0.71	20.93 ± 0.63
ER 10%	9.67 ± 0.46	12.93 ± 0.55
NF	0.00 ± 0.00	0.00 ± 0.00
NFER (containing 10% ER)	20.60 ± 1.65^ab^	19.37 ± 0.64^ab^

^a^Significantly different (*p-value<*0.05) toward ER 10% ^b^Significantly different (*p-value<*0.05) toward NF

**Table 2:** Mathematic Model of Release Kinetics

Model	Equation	R^2^
Zero-order	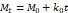	0.8792
First-order	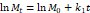	0.7413
Higuchi	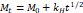	0.9503
Korsmeyer—Peppas	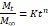	0.9644
